# Analysis of mRNA and Long Non-Coding RNA Expression Profiles in Developing Yorkshire Pig Spleens

**DOI:** 10.3390/ani11102768

**Published:** 2021-09-23

**Authors:** Xinjian Li, Xuelei Han, Caixia Sun, Gaiying Li, Kejun Wang, Xiuling Li, Ruimin Qiao

**Affiliations:** College of Animal Science and Technology, Henan Agricultural University, Zhengzhou 450046, China; lxjlongfei@163.com (X.L.); hxl014@126.com (X.H.); scx12672@126.com (C.S.); ligaiying@126.com (G.L.); wangkejun.me@163.com (K.W.); xiulingli@henau.edu.cn (X.L.)

**Keywords:** mRNA, lncRNA, developing spleen, Yorkshire pigs

## Abstract

**Simple Summary:**

Epidemic disease is a prominent problem in intensive pig production. The spleen is a blood bank and the largest immune organ, and most of the diseases in pig farms will be reflected as spleen abnormality. The results showed how the mRNA–lncRNA expression profiles in Yorkshire spleens varied with age (seven, 90, and 180 days after birth). Our study shows that 90 days after birth the gene-expression profile of pig spleen no longer changes significantly. The results are helpful for a better understanding of the transcriptome and functional genomics of spleen tissue in farm animals and could provide reference for precision pig disease research and prevention and control in pig farms.

**Abstract:**

Epidemic diseases cause great economic loss in pig farms each year; some of these diseases are characterized mainly in the spleen, but mRNA and lncRNA (long non-coding RNA) expression networks in developing Yorkshire pig spleens remain obscure. Here, we profiled the systematic characters of mRNA and lncRNA repertoires in three groups of spleens from nine Yorkshire pigs, each three aged at seven days, 90 days, and 180 days. By using a precise mRNA and lncRNA identification pipeline, we identified 19,647 genes and 219 known and 3219 putative lncRNA transcripts; 1729 genes and 64 lncRNAs therein were found to express differentially. The gene expression characteristics of genes and lncRNAs were found to be basically fixed before 90 days after birth. Three large gene expression modules were detected. The enrichment analyses of differentially expressed genes and the potential target genes of differentially expressed lncRNAs both displayed the crucial roles of up-regulation in immune activation and hematopoiesis, and down-regulation in cell replication and division in 90 days and 180 days compared to seven days. ENSSSCT00000001325 was the only lncRNA transcript that existed in the three groups. *CDK1*, *PCNA*, and *PLK* were detected to be node genes that varied with age. This study contributes to a further understanding of mRNA and lncRNA expression in different developmental pig spleens.

## 1. Introduction

Epidemic disease in farms is an outstanding problem hampering pig production. As the spleen plays a vital role in hemofiltration and antigen-specific immunity and function as a blood bank and the largest immune organ, many epidemic diseases in pig farms are associated with spleen damage, such as PRRS (Porcine Reproductive and Respiratory Syndrome), PCV (Porcine Circovirus), Pseudorabies, Swine Fever, and so on. To cope with those common epidemics, pig farms have a routine immunization program. The immunization activities of piglets usually start from seven days after birth. And in general, hogs complete their routine immunization procedures at 70 days and are on the market at about 180 days after sufficient withdrawal time. Therefore, it is of great significance to understand the time spectrum of immune system development in the spleens of domestic pigs. However, only a few reports on the RNA expression of pig spleens have been published [1,2,3,4,5,6]. 

Elucidating the development process of the spleen immune system needs an in-depth understanding of RNA expression. In addition to the mRNA that encodes the protein, as a group of non-coding RNAs with a length longer than 200 bp, lncRNA has various styles, bulky quantity, and function diversity, although their expression level is lower than that of mRNAs [7]. The lncRNAs are known to affect shearing, transduction and transcription, and degradation of mRNA through combination with mRNA after transcription and function in epigenetic regulation [8,9]. Up to 2297 causative associations between lncRNAs and diseases have been detected [10,11] (http://www.rnanut.net/lncrnadisease/, accessed on 3 April 2021). In other livestock, such as cattle and chickens, lncRNAs have also been found to be associated with some common disorders, such as mastitis [12,13], growth-retardation [12], cancer [14], and Marek’s disease in chickens [15].

The Yorkshire pig is the most widely used first dam in commercial pig production, but no temporal RNA sequencing study has been reported about Yorkshire spleens, and the likely molecular immune mechanism changes as the age increases in Yorkshire spleens remain controversial. In order to understand gene and lncRNA repertoires in Yorkshire spleens, we now appreciate the pervasive transcription of numerous mRNAs and lncRNAs in Yorkshire spleens at seven days, 90 days, and 180 days after birth in order to help us in understanding the pig immune system development better. 

## 2. Materials and Methods

### 2.1. Ethics Statement

All procedures involving animals follow the use guidelines of experimental animals established by the Ministry of Agriculture of China, the guidelines of Institutional Animal Care and Use Committee College (IACUC) of College of Animal Science and Technology of Henan Agricultural University (NO: 11-0085) and the Science Ethics Committee of Henan Agricultural University (NO: HNND20211082001).

### 2.2. Sample Collection

Spleen samples were collected from nine healthy Yorkshire pigs of both sexes, each three at the ages of seven days, 90 days, and 180 days. All the animals were self-feeding in independent cages with normal epidemic prevention. The spleen tissues from these nine pigs were collected under the same conditions, according to the requirement of total RNA extraction. 

### 2.3. Library Preparation and RNA Sequencing

The total RNA of the above nine spleen tissues was extracted using TRIZOL Reagent (Cat#15596-018, Life technologies, Carlsbad, CA, USA) following the instructions. Each specimen was purified by RNeasy micro kit (Cat#74004, QIAGEN GmBH, Dusseldorf, Germany) and RNase-Free DNase Set (Cat#79254, QIAGEN GmBH, Dusseldorf, Germany) and qualified with Nanodrop ND-2000 and Agilent Bioanalyzer 2100 (Agilent technologies, Santa Clara, CA, USA) to have RNA Integrity Numbers (RIN) of 9.8–10 and concentrations of more than 100 ng/μL. Qualified RNA samples of three individuals at the same age were equally pooled together to form three RNA groups, Y-7, Y-90, and Y-180. These three RNA groups were sent to Shanghai Biotechnology Corporation for lncRNA and mRNA sequencing by an Illumina HiSeq 2500 sequencer to produce paired reads (2 × 125 nt). 

### 2.4. RNA Mapping and Expressing Quantification 

Pair-end raw reads were filtered out to remove adaptors, low-quality reads that were shorter than 35 bases, with Q ≥ 20 base ratio less than 50% (Q = −10 log error_ratio), or bases with Q less than 10 at the 3′ end, to get clean reads with the help of Seqtk software (https://github.com/lh3/seqtk, Seqtk-1.2-r94 accessed on 3 April 2021). Trimmed clean reads were aligned to pig genome assembly (*Sus scrofa 10.2*) using TopHat (version: 2.0.13) [16] with default sets. Mapped transcripts were assembled individually with Cufflinks (version: 2.1.1) and Cuffquant [17]. The lncRNA was identified by Cuffcompare (version: 2.2.1) [18], Hmmscan [19], BLASTX and Coding Potential Calculator (CPC) [20]. Those transcripts that did not code a protein or a known protein-coding domain and with a CPC score of no more than 0 were considered as putative lncRNAs.

The RNA expression level of the genes, transcripts and lncRNAs were calculated using FPKM (fragments per kilobase of exon per million fragments mapped) [21]. The differentially expressed genes and lncRNAs were identified by fold change Fisher-test. We used a false discovery rate (FDR) of no more than 0.05 (FDR ≤ 0.05) as the significant threshold. 

### 2.5. Enrichment Analysis upon Differentially Expressed Genes and Potential Target Genes of lncRNAs

In order to evaluate the mRNA expression level and involving pathways in different development periods of Yorkshire spleens, the genes were first converted into their corresponding Homo sapiens (H. sapiens) Entrez gene IDs using the latest version of the Metascape database (https://cytoscape.org/, updated on 1 February 2021). The enrichment analysis of the GO term, the KEGG pathway, and the protein-protein interaction upon the differentially expressed mRNAs and potential targeting genes of lncRNAs were performed using Metascape. The differentially enriched terms were displayed with Heatmap. Put simply, we first identified all statistically enriched terms (GO and KEGG) and calculated the accumulative hypergeometric *p*-values and enrichment factors. The significant terms were then hierarchically clustered into a tree based on Kappa-statistical similarities among their gene memberships. Then a 0.3 kappa score was applied as the threshold to cast the tree into term clusters. The term with the best *p*-value within each cluster was selected as its representative term and we displayed them in a dendrogram. All the genes in the genome were used as the enrichment background. Terms with a *p*-value < 0.01, a minimum count of 3, and an enrichment factor > 1.5 (the enrichment factor is the ratio between the observed counts and the counts expected by chance) were collected and grouped into clusters based on their membership similarities. 

### 2.6. Gene Network Analysis

The gene expression profiles at three different developmental timings were clustered to find genes with the same expression model using STEM (Short Time-series Expression Miner) software [22]. The protein-,protein interaction (PPI) network for all the differentially expressed genes in the spleen was analyzed using STRING (https://string-db.org/, accessed on 3 December 2020) and displayed using Cytoscape (https://cytoscape.org/, accessed on 3 December 2020). Top gene networks and regulators in Y-90 vs. Y-7, Y-180 vs. Y-90 and Y-180 vs. Y-7 were obtained through IPA (http://www.ingenuity.com/products/ipa, accessed on 1 December 2020).

### 2.7. Real-Time PCR

The lncRNA and mRNA quantification was performed with using real-time PCR. The cDNA from Yorkshire spleens aged at seven days, 90 days, and 180 days were synthesized with random primers using the First Strand cDNA Synthesis Kit (Takara, Japan) according to the instructions. The PCR reaction system contained 5 μL 2× Taq Master Mix (Takara, Kyoto, Japan), 3.6 μL of sterilized distilled water, 0.2 μL forward primers (10 µM), 0.2 μL reverse primers (10 µM), and 1 μL cDNA. The PCR reaction procedure was as follows: pre-denaturation at 94 °C for 5 min, followed by 40 cycles of pre-denaturation at 94 °C for 30 s, annealing for 30 s, extension at 72 °C for 30 s, and extension for 8 min, stored at 12 °C. Primer sequences used for the RT-PCR experiments were listed in Table S1. The expression level of RNA was calculated using the 2^−ΔΔCt^ method, allowing standard error of no more than 0.2.

## 3. Results

### 3.1. Statistics of Clean Reads Alignment

After RNA sequencing and reads quality control, we obtained over 98 million 2 × 125 bp paired-end, trimmed clean reads from each group, corresponding to an average of 12.3 Gb in size, of which over 79.4% were mapped to the *Sus scrofa* 10.2 assembly genome (Table 1). Approximately, 75.3%, 36.8%, 2.4%, 24.9%, 13.5%, and 24.7% of the clean reads were mapped to gene, coding, splicing, intron, no-coding, and intergenic regions in Y-3 while 71.5%, 27.6%, 1.3%, 29.6%, 14.3%, and 28.5% were mapped in Y-90 and 71.7%, 27.9%, 1.4%, 29.3%, 14.5%, and 28.3% were mapped in Y-180 (Figure S1). After aligning, we obtained 19,647 genes in total in nine Yorkshire spleens at seven days, 90 days, and 180 days old.

### 3.2. Profile of Distinct Expressed Genes

**Figure 1:**
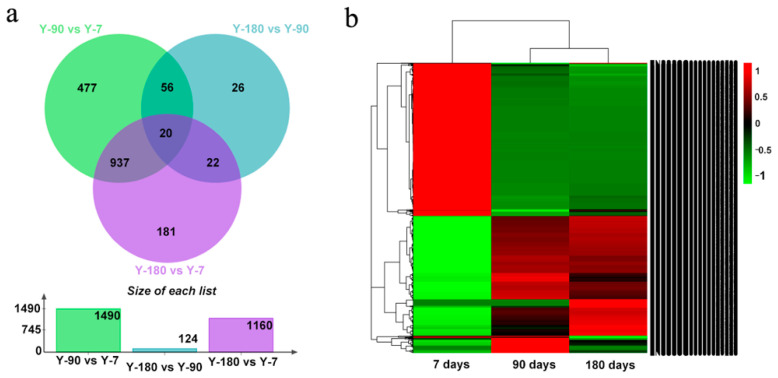
Venn diagram and heat map of 1729 differentially expressed genes. (**a**) Venn diagram; (**b**) heat map.

In order to understand how genes varied in the spleen tissues of Yorkshire pigs at different development stages, we compared the expression levels of genes in the three age groups, Y-7, Y-90, and Y-180. Our ranking analysis (see Materials and Methods) yielded a list of 1729 differentially expressed genes, among which 1490 (617 up, 837 down) genes were in Y-90 vs. Y-7; 1160 (556 up, 604 down) genes were in Y-180 vs. Y-7; and 124 (58 up, 66 down) genes were in Y-180 vs. Y-90. We found that the gene expression pattern in Y-90 and Y-180 showed a high degree of consistency (Figure 1). Of the 1160 differentially regulated genes in Y-180 vs. Y-7, 957 were also detected in Y-90 vs. Y-7. Only 27 differentially expressed genes were in common at the three stages, of which 11 genes were protein-coding genes with characters, including *IGKV-3*, *IGKV-7*, *IGKV-11*, *IGLV-2*, *IGLV-3*, *SLA-3*, *SLA-10*, *CXCL9*, *HDAC9*, *HSPA1A*, and *MRC2*. What was interesting was that some extremely differentially expressed miRNAs were detected. For example, ssc-mir-199a-2 expressed in an extremely high level in Y-7 but was almost absent in Y-90 and Y-180 (4.7E—10, 4.7E—10), whereas ssc-mir-4332 expressed in an extremely high level in Y-7 and Y-180 but was absent in Y-90 (1.1E—8). In addition, ssc-mir-451 was down-regulated both in Y-90 and Y-180 (0.2, 0.2).

### 3.3. GO and KEGG Enrichment Analysis of the Distinct Genes

**Figure 2:**
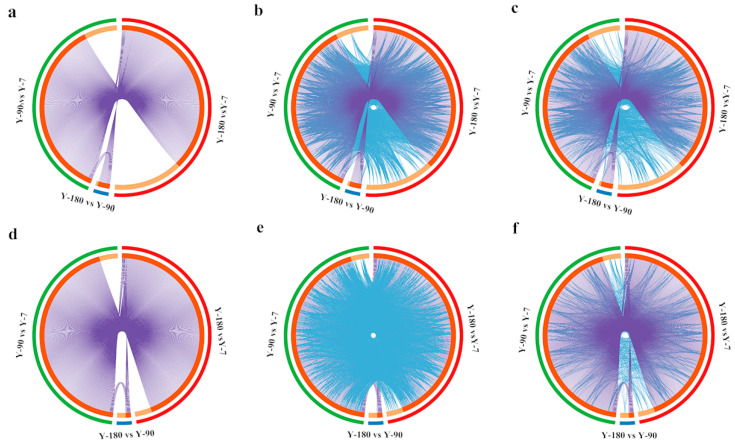
Circos plot of how 1729 differentially expressed genes overlapped among three groups. Purple curves link identical up-regulated (**a**) and down-regulated (**d**) genes at the shared gene level. At the shared term level, blue curves link up- and down-regulated genes that belong to the same enriched up-regulated (**b**) and down-regulated (**e**) GO terms and the same enriched up-regulated (**c**) and down-regulated (**f**) KEGG terms. The inner circle represents up- and down-regulated gene lists, where hits are arranged along the arc. Genes that hit multiple lists are colored in dark orange, and genes unique to a list are shown in light orange.

After converting all the genes in the pigs into their corresponding H. sapiens Entrez gene IDs, 413, 27, and 497 differentially expressed genes were remaining in Y-90 vs. Y-7, Y-180 vs. Y-90, and Y-180 vs. Y-7. The GO and KEGG enrichment analysis of these differentially expressed genes were performed using Cytoscape. The overlapped up- and down-regulated genes and genes that shared the same enriched GO and KEGG ontology term(s) were shown in the Circos plots (Figure 2). Both the up- and down-regulated gene expression profiles in Y-90 and Y-180 showed great similarity. Comparing with the up-regulated genes, only a small number of the down-regulated genes that colored in light orange were unique to each of the three groups of samples.

**Figure 3:**
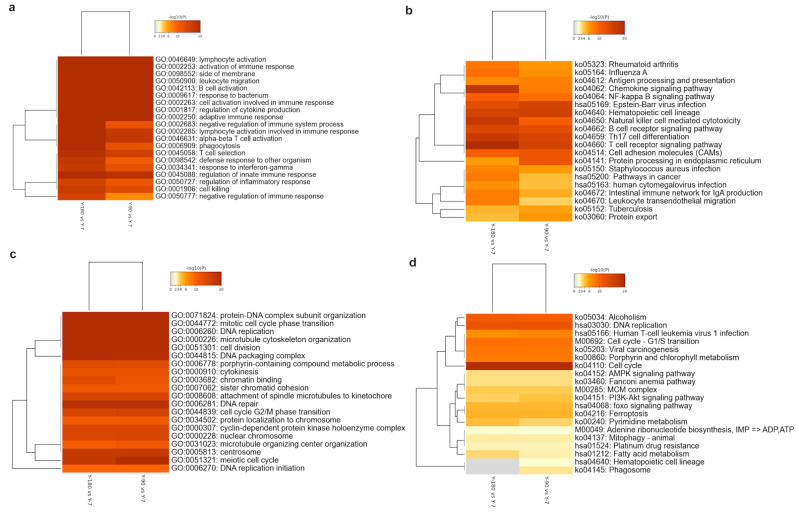
Heatmap of top 20 enriched up- and down-regulated GO terms and KEGG pathways. Up-regulated GO (**a**) and KEGG (**b**), down-regulated GO (**c**) and KEGG (**d**). The heatmap cells are colored by their *p*-value; white cells indicate the lack of enrichment for that term in the corresponding gene list.

In order to understand these enriched GO and KEGG ontology terms in detail, we extracted the top 20 up- and down-regulated GO and KEGG clusters in the three groups and displayed them in the heatmaps (Figure 3). No significant up- or down-regulated enriched term was found in Y-180 vs. Y-90. As the age increased, various GO terms related to the immune system were accumulated in large numbers (Figure 3a), such as GO: 0046649 and GO: 0002253, whereas the significant down-regulated genes were almost enriched in cell replication and division (Figure 3c), such as GO: 0006260 and GO: 0051301. For the up-regulated genes-enriched GO terms, 14 out of the top 20 had almost equally significant levels in both Y-90 vs. Y-7 and Y-180 vs. Y-7, among which the activation of lymphocytes and B cells, and the regulation of various immune responses were at a relatively higher significant level. The other six GO terms of the top 20 all had a more significant level in Y-180 vs. Y-7 than in Y-90 vs. Y-7, which were enriched in negative regulation of immune response and phagocytosis, such as GO: 0050777 (negative regulation of the immune response) and GO: 0002683 (negative regulation of the immune system process). For the down-regulated GO terms, 18 of the top 20 were at almost the same level in Y-180 and Y-90. 

With the age increased, disease infection and immune cell signaling pathways were up-regulated in both Y-90 vs. Y-7 and Y-180 vs. Y-7 (Figure 3b). But almost all the up-regulated pathways had different enrichment levels in Y-90 vs. Y-7 and Y-180 vs. Y-7. The enrichment of chemokines, natural killer cells, T cells, and other immune cells and intestinal immunity were much higher in Y-180. Cell cycle, alcoholism, and DNA replication pathways were highly down-regulated in Y-90 vs. Y-7 and Y-180 vs. Y-7 with almost the same enrichment levels in the two groups (Figure 3d). Signaling pathways including AMPK, mitophagy, adenine ribonucleotide biosynthesis, pyrimidine metabolism, insulin related PI3K-Akt and FOXO, anemia, ferroptosis, and fatty acid metabolism were less down-regulated. The down-regulation of pyrimidine and fatty acid metabolism were higher in Y-180 vs. Y-7 while the PI3K-Akt signaling pathway was higher in Y-90 vs. Y-7. Hematopoietic cell lineage and phagosome were only found in Y-90 vs. Y-7.

### 3.4. Network Analysis of Known Differentially Expressed Genes 

**Figure 4:**
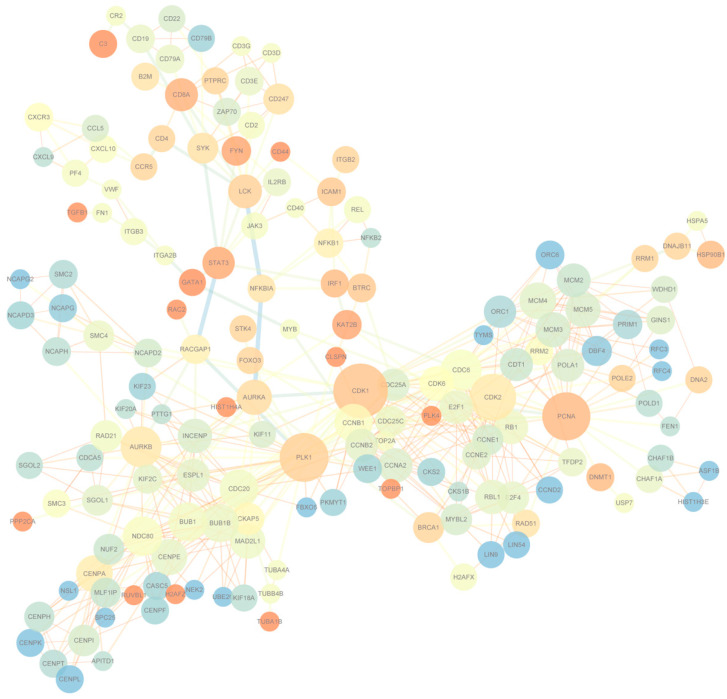
Protein-protein interaction network of differentially expressed module genes.

To understand the differentially expressed gene patterns and the gene interactions involved in the significant expression modules at seven days, 90 days, and 180 days after birth in the Yorkshire spleens, we first used STEM to perform gene expression model analysis on the 1729 genes and the results are shown in Figure S2. A total of eight modules were obtained, of which three were significant and contained 1175 genes. Within the three significant modules, one included 666 genes that were up-regulated from seven days to 90 days and maintained the regulation in 180 days; the other one involved 850 genes that were down-regulated from seven to 90 days and maintained the regulation in 180 days; and the last one contained 52 genes that were up-regulated from seven days to 90 days and up-regulated from 90 days to 180 days. Then, we used STRING to conduct PPI analysis on the 1175 module genes. A total of 756 interactions with combined scores of no less than 0.97 were filtered to display using Cytoscape. Of the networks that combined no less than three edges, having an average shortest path length of no less than three and with no less than three neighbors within distance, two are finally displayed in Figure 4. There were 165 nodes and the center nodes genes were *CDK1*, *PCNA*, and *PLK* in the Yorkshire spleen gene network.

**Figure 5:**
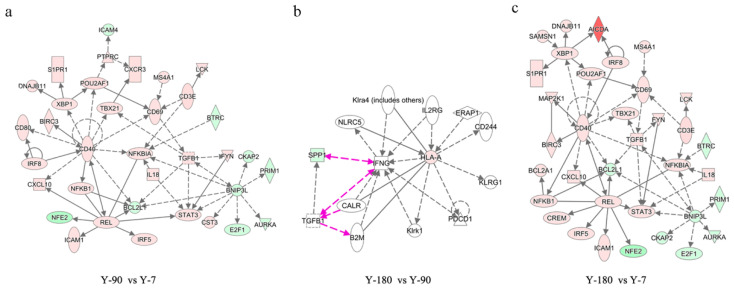
Top significant IPA network of differentially expressed genes in three groups. Top significant IPA network of differentially expressed genes in Y-90 vs Y-7 (**a**), Y-180 vs Y-90 (**b**) and Y-180 vs Y-7 (**c**).

To further capture how the relationships of the functional genes varied from one week to 180 days after birth, differentially expressed genes in Y-90 vs. Y-7, Y-180 vs. Y-90, and Y-180 vs. Y-7 were selected to analyze network enrichment using IPA under default settings. The significant enriched networks of differentially expressed genes further supported the view of the similarity between Y-90 and Y-180 groups. The top significant gene networks of Y-90 vs. Y-7, Y-180 vs. Y-7, and Y-180 vs. Y-90 are exhibited in Figure 5. These networks were related to cellular function and maintenance; hematological system development and function; and cell death and survival. The center genes of the networks of Y-90 vs. Y-7 and Y-180 vs. Y-7 were almost the same, such as *CD40*, *REL*, and *BNIP3L*. Only *SPP1* and *HLA-A* were involved in the network of Y-180 vs. Y-90. In addition to that, the top regulators in the three groups were obtained, including BNIP3L, IL5, CD38, TGFβ1, and BCL3 in Y-90 vs. Y-7; BNIP3L, IL5, CD38, TGFβ1, and IL4 in Y-180 vs. Y-7; and ERAP1, NLRC5, and IL2RG in Y-180 vs. Y-90.

### 3.5. Expression Profile of LncRNAs 

After genome aligning, transcript assembling, and FPKM calculating (see Materials and Methods), we detected in total 219 known and 3291 putative lncRNAs, including 22 exonic_sense, 294 exonic_antisense, 232 intronic_sense, 232 intronic_antisense, 2435 intergenic, and 295 bidirectional lncRNAs (Figure S3a). These lncRNAs were further classed into 2712 cis_lncRNAs and 377 trans_lncRNAs, of which 294 were in common. The 2712 cis and 377 trans lncRNAs were predicted to target 1945 and 203 genes, of which 1108 and 116 genes were uniquely annotated. The average function distance for cis and trans lncRNA was about 2.50 kb and 89.29 kb.

### 3.6. GO and KEGG Enrichment Analysis of lncRNAs Target Genes

For the cis and trans lncRNA target genes, the top 20 GO and KEGG terms enrichment analyses were carried out with GO Biological Processes, GO Cellular Components, and GO Molecular Functions ontology sources and KEGG Functional Sets, KEGG Pathway, and KEGG Structural Complexes ontology sources under default settings. All the genes in the genome were used as the enrichment background. 

**Figure 6:**
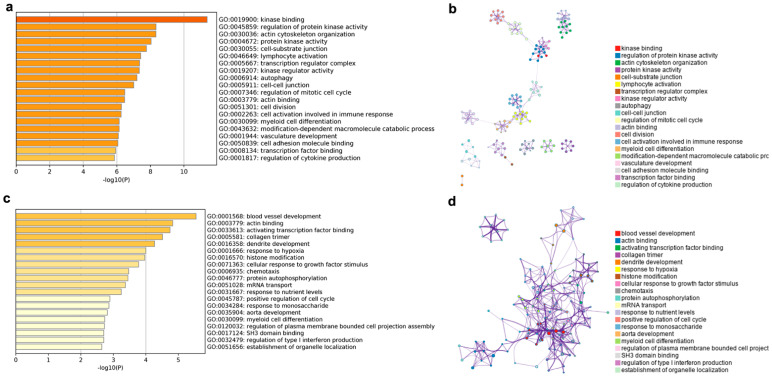
Top 20 enriched GO terms and networks involved in cis and trans lncRNA target genes. GO terms of cis lncRNA (**a**) and trans lncRNA (**c**), colored by *p*-values. GO terms networks of cis lncRNA (**b**) and trans lncRNA (**d**), colored by GO cluster ID, where nodes that shared the same cluster ID were typically close to each other.

The enriched GO terms and GO term networks of the cis lncRNA target genes are shown in Figure 6a,b. From Figure 6a, the GO terms were mainly enriched in six biological processes, including kinase, cytoskeleton, transcription regulator, immunity, cell cycle, and vasculature development. Among them, the GO terms including kinase, immunity, cell cycle, and vasculature development clustered together to form eight subnetworks, which were linked to each other to form the largest network (Figure 6b). 

The target genes of the trans lncRNA were enriched in the same two terms as the cis lncRNA in actin binding (GO: 0003779) and myeloid cell differentiation (GO: 0030099) (Figure 6c). Meanwhile, a large number of the enriched terms were similar to those of the cis lncRNA target genes, such as blood vessel development, activating transcription factor binding, collagen trimmer, chemotaxis, and protein autophosphorylation. In addition, some of the new terms, such as dendrite development, response to hypoxia, cellular response to growth factor stimulus, and response to nutrient levels and monosaccharide were also enriched for the trans lncRNA target genes. With the exception of mRNA transport, all the terms were tightly linked to each other in a network (Figure 6d), unlike the obvious differentiation of single subnetworks in the cis lncRNA target genes.

**Figure 7:**
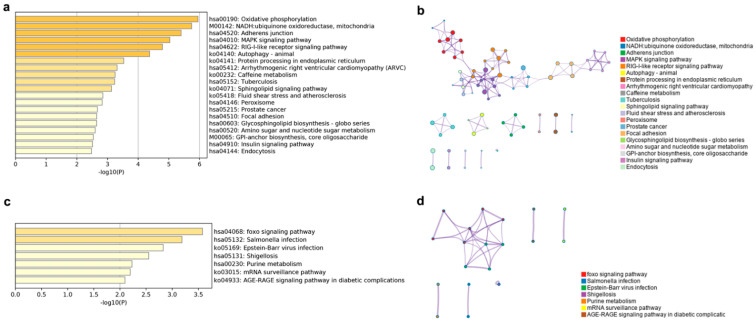
Top 20 enriched KEGG terms and KEGG networks involved in cis and trans lncRNA target genes. KEGG terms of cis lncRNA (**a**) and trans lncRNA (**c**), colored by *p* values. KEGG terms networks of cis lncRNA (**b**) and trans lncRNA (**d**), colored by KEGG cluster ID, where nodes that share the same cluster ID are typically close to each other.

The putative cis lncRNA target gene enriched KEGG terms were mainly related to oxidative phosphorylation, NADH, the MAPK and RIG-I like receptor signaling pathway, and so on (Figure 7a). The oxidative phosphorylation, the MAPK signaling pathway, the RIG-I-like receptor signaling pathway, the autophagy-animal, the NADH-ubiquinone oxidoreductase, the mitochondria, the focal adhesion, and the arrhythmogenic right ventricular cardiomyopathy subnetworks were linked to form the largest KEGG network (Figure 7b). Target genes of putative trans lncRNA had an enrichment in seven KEGG terms, mainly in the FOXO signaling pathway, immune infection, purine and sugar metabolism, and the mRNA surveillance pathway (Figure 7c). The FOXO signaling pathway and the Epstein-Barr virus infection terms were connected into a network (Figure 7d).

### 3.7. Differentially Expressed Profile of LncRNAs 

Using the same criteria as for mRNA, our ranking analysis yielded a list of 64 differentially expressed lncRNAs that had a RPKM value of more than one, among which 51 genes (16 up, 35 down) were in Y-90 vs. Y-7; nine genes (4 up, 5 down) were in Y-180 vs. Y-90; and 56 genes (20 up, 36 down) were found in Y-180 vs. Y-7. We found that the lncRNA expression pattern in Y-90 and Y-180 also showed a high degree of consistency. Of the 51 differentially regulated lncRNAs in Y-90 vs. Y-7, 43 were also found in Y-90 vs. Y-7. This similarity for genes between Y-90 and Y-180 also existed for lncRNAs. The 64 differentially expressed lncRNAs were located on 13 genes, of which only three encode proteins with characters, *SLA-3*, *SLA-8*, and *SLA-9*. *SLA-3* was up-regulated in Y-90 vs. Y-7 and Y-180 vs. Y-7, then down-regulated from the age of 90 days to 180 days. *SLA-8* was up-regulated in Y-180 vs. Y-7. *SLA-9* was up-regulated in Y-180 vs. Y-7 and Y-180 vs. Y-90. The only differentially expressed lncRNA transcript for the three groups in common was ENSSSCT00000001325 on the *SLA-3* gene.

In order to understand the characteristics of these 64 differentially expressed lncRNAs in Yorkshire spleen tissues at the three time points, and the biological processes involved, we predicted the target genes of the 64 lncRNAs and carried out KEGG pathway enrichment analyses of those genes at each time point. In Y-90 vs. Y-7, four target genes were enriched in six pathways, including RRP7A in hsa03008 (ribosome biogenesis); BMP6 in hsa04340 and hsa04350 (hedgehog and TGF-β signaling pathway); NUP205 in hsa03013 (RNA transport); and DDO in hsa00250 and hsa04146 (alanine, aspartate and glutamate metabolism and peroxisome). In Y-180 vs. Y-7, except for the three same target genes, RRP7A, BMP6, and DDO, and the five same pathways involved in Y-90 vs. Y-7, two new target gene POLR2D functions in five pathways, including hsa00240 (pyrimidine metabolism); hsa00230 (purine metabolism); hsa03020 (RNA polymerase); etc., and COL5A2 functions in four pathways, including hsa04512 (ECM receptor interaction); hsa04974 (protein digestion and absorption); hsa04510 (focal adhesion); and hsa05146 (amoebiasis) were detected.

Taking advantage of the mRNA expression profile in this study, we further analyzed the potential target differentially expressed genes of the 64 lncRNAs (Table S2). More than one lncRNA was found to target more than one gene. As a result, 32 of the 64 lncRNAs targeted 31 differentially expressed Ensembl genes, of which 23 genes had characters. In detail, 24, 24, and nine lncRNAs potentially targeted 24, 21, and 6 Ensembl genes in Y-90 vs. Y-7, Y-180 vs. Y-7, and Y-180 vs. Y-90, of which 18, 21, and 4 genes had characters. Within the 23 characteristic genes, 17 were overlapped in Y-90 vs. Y-7 and Y-180 vs. Y-7 and with the same up- down-regulated state. The *SLA-3* and *SLA-8* genes were acquired immunity related genes; the *SCARNA2* and SCARNA6 genes were members of a small Cajal body-specific RNA family; NCAPD3 (Non-SMC Condensin II Complex Subunit D3) is a subunit of condensin II, a large protein complex involved in chromosome condensation; BMP6 (Bone morphogenetic protein 6) is a member of the TGFβ gene coding protein; ANP32B (Acidic Nuclear Phosphoprotein 32 Family Member B) functions in RNA polymerase binding; and AHSP (Alpha-hemoglobin-stabilizing protein) is involved in hemoglobin assembly. 

### 3.8. Real-Time PCR 

**Figure 8:**
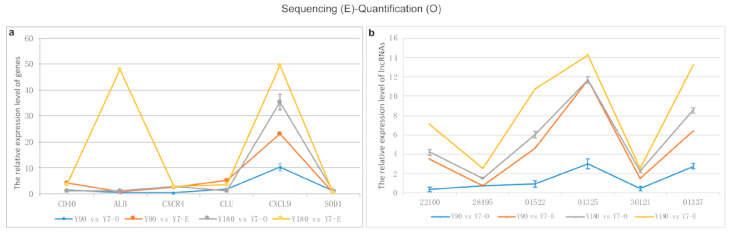
Comparison of RNA sequencing and RT-PCR results for six genes and six lncRNA transcripts. (**a**) gene; (**b**) lncRNA transcripts (ENSSSCT00000001522, −01325, −22100, −30121, −28495).

In total, six differentially expressed genes and six LncRNA transcripts were randomly selected to perform RT-PCR in order to validate the sequencing data using *GAPDH* as the internal control gene, respectively. The sequencing and experimental results of these genes and lncRNAs are presented in Figure 8. It was shown that there was a relatively good consistency between the sequencing and quantitative experimental results, except for the *ALB* gene and the lncRNA transcript ENSSSCT00000022100. 

## 4. Discussion

As the largest immune organ, the spleen is the center of cellular immunity and the humoral immunity of humans that plays an important role in disease resistance and hematopoiesis. Similarly, the spleen is an important peripheral lymphatic organ of pigs that is involved in physiological functions, such as hematopoiesis, blood filtration, blood storage, and the immune response of the body. Its structure and function are gradually developed during the growth and development of pigs. However, only one report on Chinese indigenous and wild pig spleen lncRNAs has been reported. Here we selected nine large Yorkshires aged at seven days, 90 days and 180 days, and analyzed the transcriptional expression of the lncRNA and mRNA in their spleen tissues for the first time. This is the first study on lncRNA–mRNA in the spleens of commercial pigs.

We identified 19,647 genes and 3438 lncRNA transcripts in the nine spleen tissues, among which 1729 genes and 64 lncRNAs were found to be differentially expressed among the three development stages. Compared with the genes, most of the lncRNAs that were detected were new lncRNAs and the differentially expressed lncRNA transcripts were much fewer. On the other hand, the lncRNA of spleen tissue did not change much with time and was relatively conservative between different development stages in pigs. 

We found significant dynamically expressed genes and lncRNAs between both 7-day-old and 90-day-old splenic tissues, but no dramatic differences between 90-day-old and 180-day-old splenic tissues. Combined with the fact that there was no obvious difference in gene expression between 0-day-old and 30-day-old, and 30-day-old and 180-day-old Rongchang pig spleens [1], it suggested that the period from 30 days to 90 days of age might be the developmental stage for gene expression changes in the spleens of pigs, and there was little change in spleen development during the whole lactation stage. This is a bit different from the idea that the immune system would build up in the first week of life in pigs. 

We found a certain degree of consistent biological function between protein-coding genes and the target genes of lncRNAs, especially for the target genes of cis lncRNAs. From seven days to 90 days after birth, the expression of genes and target genes of lncRNA in GO and KEGG pathways associated with the immune system was increased, while the expression of genes associated with cell replication and division was decreased. This dynamic transcriptional change well reflected the biological function of the spleen. Moreover, it suggested that the function of the spleen in T cell and lymphocyte activation was not species-specific in farm animals [3], and that the changing of gene expression with age was relatively conservative among pig breeds [1]. 

The GO and KEGG enriched terms of down-regulated genes showed higher consistency between Y-90 vs. Y-7 and Y-180 vs. Y-7. And for the GO terms of up-regulated genes, only the enrichment degree of the negative regulation of the immune system process, the negative immune response, and phagocytosis were much higher at 180 days. But almost all the enriched pathways of up-regulated genes had different enriched levels between 90 days and 180 days except for the B cell signal receptor pathway.

We also found that there were differences in the enrichment of lncRNA target genes and mRNAs. Both the cis and trans target genes of lncRNA were concentrated in the transcription factor binding and transcription factor complex GO terms, which were corresponding to the action mode of the lncRNAs. In addition, we found that the functions of the lncRNA target genes seemed to be more diverse compared to the mRNAs, and that the functions of cis and trans target genes have different priorities. In general, the enrichment of the cis target genes was more diverse and with a much higher degree. For the GO terms, the cis target genes were mainly enriched in kinases in addition to transcription factor binding, immunity, and cell division, while the trans target genes were mainly enriched in actin binding, dendrites, nutrition, and growth and development factor stimulation in addition to blood vessel development, activating transcription factor binding, and positive regulation of cell cycle and chemotaxis. For the KEGG pathways, the cis target genes were mainly concentrated in oxidative phosphorylation, NADH, MAPK, immunity, glucose metabolism, and lipid metabolism, while the trans target genes were enriched in FOXO, immunity, glucose metabolism, etc. This suggested that the regulation of lncRNA on spleen development may be more detailed and complex.

## 5. Conclusions

In summary, our study provided the mRNA–lncRNA profiles in Yorkshire spleens at three developmental stages for the first time. We confirmed that the functions of lncRNA and mRNA in the spleen at different developmental stages were consistent to a certain extent, and that there might be little difference between different pig species. It was speculated that 30–90 days after birth was an important stage for the change of spleen gene expression. Compared with mRNA, the regulation of lncRNA on spleen development may be more complex. To understand the regulation on developing spleens more clearly, more study should be added on the period between 30 days and 90 days after birth.

## Figures and Tables

**Table 1:** Genomic region distribution of the output of clean reads.

Group	Total Clean Reads	Total Mapped Reads	Unique Mapped	Region Distribution					
				Gene	Intergenic	Coding	Splicing	Intron	Non-Coding ^1^
Y-7	123,134,202	98,937,321	85,814,748	74,475,278	24,462,043	36,445,772	2,343,397	24,659,691	13,369,815
Y-90	98,709,782	78,279,022	68,422,219	55,965,083	22,313,939	21,591,787	1,048,206	23,152,959	11,220,337
Y-180	129,080,634	102,646,848	89,742,389	73,586,197	29,060,651	28,632,107	1,426,424	30,036,857	14,917,233

^1^ Non-coding includes 5′UTR, 3′UTR and noncoding RNA regions.

## Data Availability

All the sequencing data has been uploaded in GenBank with the accession number PRJNA719591.

## Supplementary Materials

The following are available online at https://www.mdpi.com/article/10.3390/ani11102768/s1, Figure S1: genomic distribution of the output of clean reads from RNA sequencing, Figure S2: eight time-series modules that detected from 1729 differentially expressed genes, Figure S3: genomic distribution of all lncRNA transcripts and Venn diagrams of 64 differentially expressed lncRNA transcripts, Table S1: primers used for quantitative PCR experiments, Table S2: potential targeted differentially expressed genes of the 64 differentially expressed lncRNA transcripts.
