# Supplementary material for: Analysis of mRNA and Long Non-Coding RNA Expression Profiles in Developing Yorkshire Pig Spleens
Source: Animals (Basel). 2021 Sep 23;11(10):2768. doi: 10.3390/ani11102768 (PMC8532824; doi:10.3390/ani11102768)
Supplement: Supplementary file 1 [file animals-11-02768-s001.zip › Table S1.pdf]

Table S1. Primers used for quantitative PCR experiments.

LncRNA	Forward（5'-3'）	Reverse（5'-3'）	Product length (bp)
ENSSSCT00000001325	CGCGGGTACAGTCAGTTTG	AGCGTGTTATTCCCCATCTCC	206
ENSSSCT00000022100	CGATGTCACAGTCCCTTCCC	CCATGACCCCACACACATGA	88
ENSSSCT00000028495	CCTTCCCTGCCCTCAA	CCGCTCCTCAGTCTCCT	180
ENSSSCT00000001337	GAAGCCAATCAAGTCACCGC	ACTGCGTGTCGTCCACATAG	243
ENSSSCT00000030121	TGTAGAGCACCCGAAACC	CACAGGCAACACCAAGAG	78
ENSSSCT00000001522	TCGTTGCTGGTCTGGTTCTC	GACTTGCGGGAGACACATCA	138
Gene	Forward（5'-3'）	Reverse（5'-3'）	Product length (bp)
SOD1	AAACATGGTGGGCCAAAGGA	TTTCCACCTCTGCCCAAGTC	185
CD40	TCCAGCGAATTCCTAGCCAC	CAGAAACCTCTGTCGCCATCT	229
CLU	AGAGCTCCAGGAAATGTCCAC	CCCTTCAGCTTTGTCTCGGT	204
CXCR4	ACGGGTTCCGTATATTCACTTCAG	CCCATGACCAGGATGACCAAT	190
CXCL9	TCAGTGGAACACCTACAGAAGTG	TCATCCTTTGGCTGGTGTTGA	160
ALB	TCCACAAGGAATGCTGCCAT	TCAGGTCTGCAGGCAATTCAT	186
GAPDH	ACGTGTCGGTTGTGGATCTG	AAGTGGTCGTTGAGGGCAAT	205
