# Supplementary material for: Analysis of mRNA and Long Non-Coding RNA Expression Profiles in Developing Yorkshire Pig Spleens
Source: Animals (Basel). 2021 Sep 23;11(10):2768. doi: 10.3390/ani11102768 (PMC8532824; doi:10.3390/ani11102768)
Supplement: Supplementary file 1 [file animals-11-02768-s001.zip › Table S2.pdf]

Table S2. Potential targeted differentially expressed genes of the 64 differentially expressed lncRNA transcripts.

**Y-90 vs Y-7**	**Diff_target gene**	**Diff_target gene symbol**
ENSSSCT00000001325	ENSSSCG00000001227	TMP-SLA-3
ENSSSCT00000001326	ENSSSCG00000001227	TMP-SLA-3
ENSSSCT00000001337	ENSSSCG00000001396	SLA-8
ENSSSCT00000019857	ENSSSCG00000018262	SNORA53
ENSSSCT00000019904	ENSSSCG00000018309	SNORD17
ENSSSCT00000020277	ENSSSCG00000018682	SCARNA2
ENSSSCT00000020729	ENSSSCG00000019134	7SK
ENSSSCT00000022100	ENSSSCG00000020505	SCARNA6
ENSSSCT00000027120	ENSSSCG00000025419	SNORA73
ENSSSCT00000028495	ENSSSCG00000030376	U1
ENSSSCT00000028495	ENSSSCG00000026740	U3
ENSSSCT00000034061	ENSSSCG00000001228	TMP-CH242-74M17.5
ENSSSCT00000034907	ENSSSCG00000030790	TMP-CH242-74M17.6
TCONS_00315059	ENSSSCG00000001027	BMP6
TCONS_00003833	ENSSSCG00000004750	-
TCONS_00391850	ENSSSCG00000021943	-
TCONS_00391850	ENSSSCG00000017579	-
TCONS_00190134	ENSSSCG00000016475	TRBC1
TCONS_00306779	ENSSSCG00000001027	BMP6
TCONS_00186792	ENSSSCG00000016475	TRBC1
TCONS_00228960	ENSSSCG00000025858	-
TCONS_00228961	ENSSSCG00000025858	-
TCONS_00350236	ENSSSCG00000022192	NCAPD3
TCONS_00004466	ENSSSCG00000005372	ANP32B
TCONS_00315059	ENSSSCG00000027411	-
TCONS_00228979	ENSSSCG00000022403	AHSP
TCONS_00391850	ENSSSCG00000029074	-
TCONS_00306779	ENSSSCG00000027411	-
TCONS_00020475	ENSSSCG00000004399	SLC22A16
**Y-180 vs Y-7**	**Diff_target gene**	**Diff_target gene symbol**
ENSSSCT00000001325	ENSSSCG00000001227	TMP-SLA-3
ENSSSCT00000001337	ENSSSCG00000001396	SLA-8
ENSSSCT00000019857	ENSSSCG00000018262	SNORA53
ENSSSCT00000019904	ENSSSCG00000018309	SNORD17
ENSSSCT00000020277	ENSSSCG00000018682	SCARNA2
ENSSSCT00000020729	ENSSSCG00000019134	7SK
ENSSSCT00000022100	ENSSSCG00000020505	SCARNA6
ENSSSCT00000034061	ENSSSCG00000001228	TMP-CH242-74M17.5
ENSSSCT00000034907	ENSSSCG00000030790	TMP-CH242-74M17.6

TCONS_00315059	ENSSSCG00000001027	BMP6
TCONS_00190134	ENSSSCG00000016475	TRBC1
TCONS_00306779	ENSSSCG00000001027	BMP6
TCONS_00186792	ENSSSCG00000016475	TRBC1
TCONS_00350236	ENSSSCG00000022192	NCAPD3
TCONS_00004466	ENSSSCG00000005372	ANP32B
ENSSSCT00000001335	ENSSSCG00000001397	TMP-CH242-74M17.4
ENSSSCT00000001522	ENSSSCG00000001396	SLA-8
ENSSSCT00000001522	ENSSSCG00000024161	SLA-7
ENSSSCT00000024114	ENSSSCG00000022489	SNORA73
ENSSSCT00000024682	ENSSSCG00000023048	Metazoa_SRP
ENSSSCT00000025491	ENSSSCG00000023855	U3
ENSSSCT00000025491	ENSSSCG00000017645	TEX14
ENSSSCT00000034266	ENSSSCG00000001397	TMP-CH242-74M17.4
ENSSSCT00000035587	ENSSSCG00000001227	TMP-SLA-3
TCONS_00228979	ENSSSCG00000022403	AHSP
TCONS_00020475	ENSSSCG00000004399	SLC22A16
**Y-180 vs Y-90**	**Diff_target gene**	**Diff_target gene symbol**
ENSSSCT00000001325	ENSSSCG00000001227	TMP-SLA-3
ENSSSCT00000001335	ENSSSCG00000001397	TMP-CH242-74M17.4
ENSSSCT00000024114	ENSSSCG00000022489	SNORA73
ENSSSCT00000025491	ENSSSCG00000023855	U3
ENSSSCT00000030121	ENSSSCG00000028335	U3
ENSSSCT00000030121	ENSSSCG00000026740	U3
ENSSSCT00000034266	ENSSSCG00000001397	TMP-CH242-74M17.4
ENSSSCT00000035587	ENSSSCG00000001227	TMP-SLA-3
ENSSSCT00000001326	ENSSSCG00000001227	TMP-SLA-3
ENSSSCT00000028495	ENSSSCG00000028335	U3
ENSSSCT00000028495	ENSSSCG00000026740	U3
