# Supplementary material for: Analysis of mRNA and Long Non-Coding RNA Expression Profiles in Developing Yorkshire Pig Spleens
Source: Animals (Basel). 2021 Sep 23;11(10):2768. doi: 10.3390/ani11102768 (PMC8532824; doi:10.3390/ani11102768)
Supplement: Supplementary file 1 [file animals-11-02768-s001.zip › Table S3.pdf]

Table S3. A list of top 10 up and down regulated protein coding genes with characters.

Y-90 vs Y-7		Y-180 vs Y-7		Y-180 vs Y-90	
Gene	Fold change	Gene	Fold change	Gene	Fold change
HBB	3.8E+8	HBB	4.3E+8	LDLR	3.1E+6
IGLV-4	2.2E+6	IGLV-4	3.7E+6	ZFX	2.5E+6
KLRD1	1.5E+6	LDLR	3.1E+6	ALB	6.2E+1
IGLV-10	1.4E+3	ZFX	2.5E+6	GP1BB	2.9E+1
IGLV-9	5.8E+2	AICDA	1.6E+6	MRC2	6.5E+0
IGLV-11	5.3E+2	KLRD1	1.6E+6	SLA-9	3.2E+0
IGLV-7	5.0E+2	IGLV-10	1.0E+4	SLA-10	3.0E+0
IGLV-8	2.2E+2	IGLV-9	7.9E+2	CXCL9	2.1E+0
IGKV-7	2.2E+2	IGLV-11	5.5E+2	/	/
IGJ	2.1E+2	IGLV-7	5.2E+2	/	/
MS4A8B	6.0E-8	KLF1	4.7E-8	NMB	6.8E-7
TSPO2	1.8E-7	SPTA1	5.6E-8	SPP1	8.6E-2
ART5	2.5E-7	MS4A8B	6.0E-8	IGLV-2	2.7E-1
CCDC42	4.6E-7	SLC22A16	9.3E-8	HSPA1A	3.5E-1
TCN1	6.2E-7	ADD2	1.4E-7	HDAC9	3.7E-1
TRIM50	6.5E-7	TSPO2	1.8E-7	IGKV-7	4.0E-1
FAM150A	6.9E-7	TNNI1	1.9E-7	MARCO	4.0E-1
PKLR	7.1E-7	ART5	2.5E-7	HSPH1	4.1E-1
PDIA2	8.0E-7	TCN1	6.2E-7	SLA-3	4.1E-1
HOXA1	9.2E-7	TRIM50	6.5E-7	IGKV-3	4.2E-1
