# Supplementary figures and images for: Analysis of mRNA and Long Non-Coding RNA Expression Profiles in Developing Yorkshire Pig Spleens
Source: Animals (Basel). 2021 Sep 23;11(10):2768. doi: 10.3390/ani11102768 (PMC8532824; doi:10.3390/ani11102768)

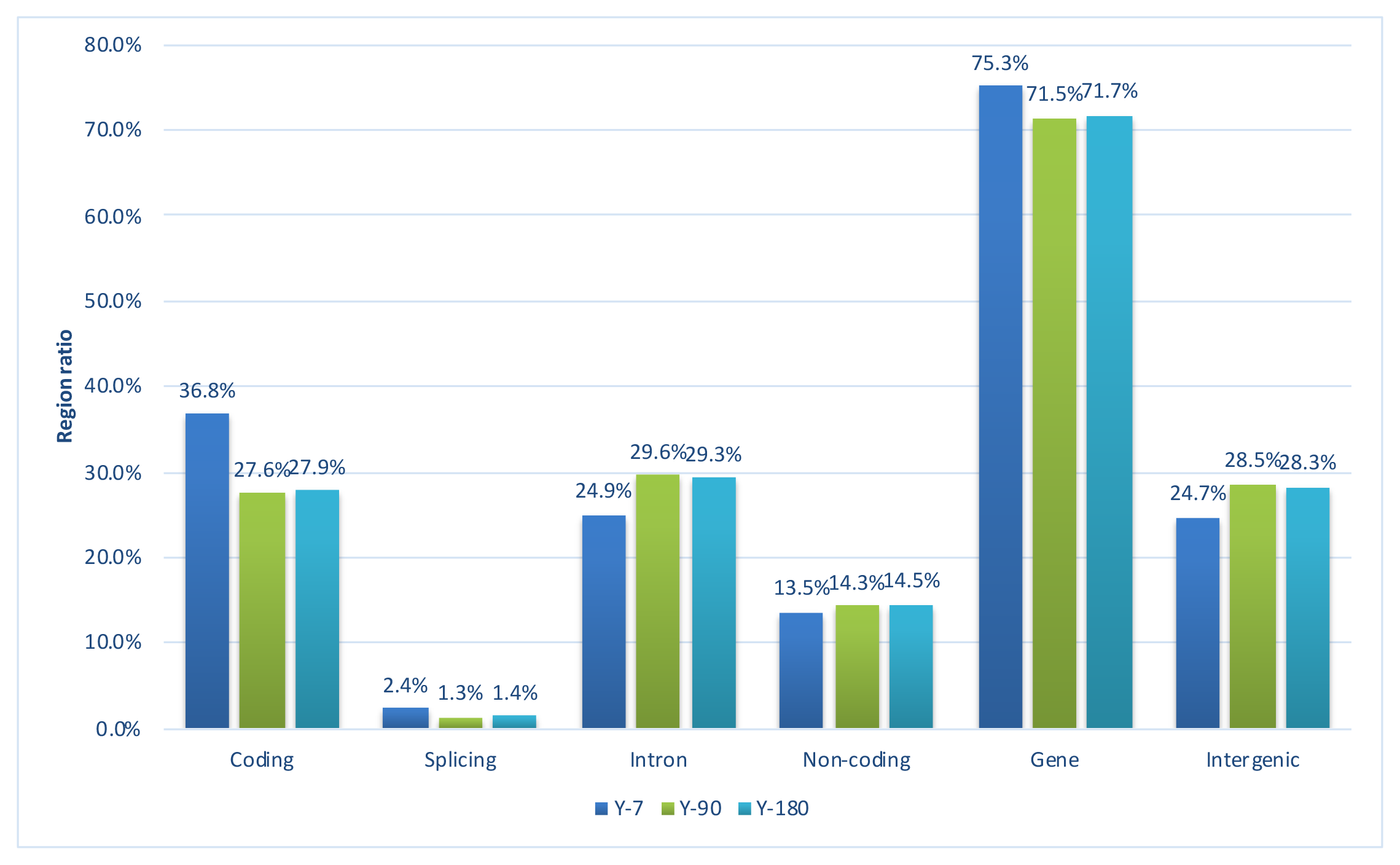

Supplement: Supplementary file 1 [file animals-11-02768-s001.zip › Figure S1.tif]

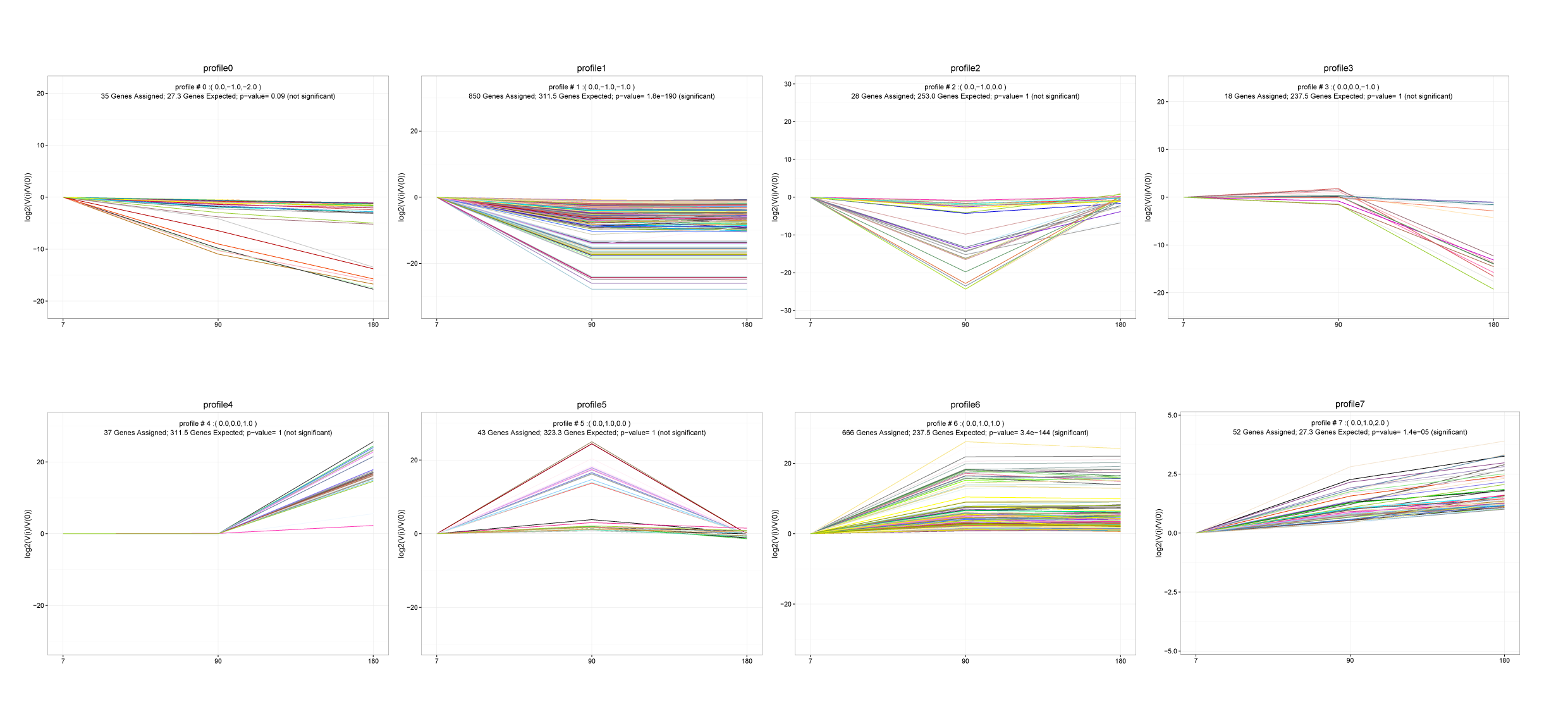

Supplement: Supplementary file 1 [file animals-11-02768-s001.zip › Figure S2.tif]

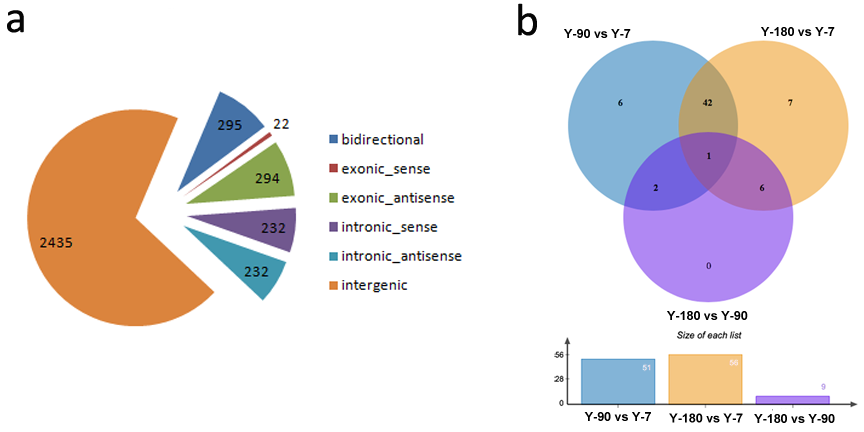

Supplement: Supplementary file 1 [file animals-11-02768-s001.zip › Figure S3.tif]
